# De Novo high-volume metastatic prostate cancer with primary resistance to standard systemic therapy and exceptional response to PSMA-Lu177: A case report

**DOI:** 10.1007/s13691-025-00834-2

**Published:** 2025-12-12

**Authors:** Desiree Rafizadeh, D. Kaakour, S. Sun, J. Herman, T. Kain, A. Rezazadeh Kalebasty

**Affiliations:** 1 University of California, Irvine School of Medicine, 1001 Health Sciences Road, 92697-4089 Irvine, CA USA; 2 Division of Hematology and Medical Oncology, University of California, Irvine Medical Center, Orange, CA USA; 3 Department of Radiology, University of California, Irvine Medical Center, Orange, CA USA; 4 Department of Medicine, University of California, Irvine Medical Center, Orange, CA USA; 5 Department of Urology, University of California, Irvine Medical Center, Orange, CA USA

**Keywords:** mCRPC, PSMA-Lu177, PSA

## Abstract

Prostate specific membrane antigen (PSMA) Lutetium-177 (Lu-177) therapy is a relatively new theranostic treatment using a targeted radioligand therapy in the treatment of metastatic prostate cancer. PSMA-Lu177 has been approved by the FDA for treatment of prostate cancer in the metastatic castrate resistant setting. Here, we present the case of a 90-year-old man with metastatic castrate resistant prostate cancer (mCRPC) and primary resistance to androgen deprivation, second-generation androgen receptor inhibitors, and docetaxel chemotherapy. Despite the lack of response to prior lines of therapy, the patient demonstrated a rapid response to PSMA-Lu177 therapy with a substantial drop in PSA level, starting from the first cycle.

## Introduction

Prostate carcinoma (PCa) is one of the most common malignancies affecting men worldwide, with a projected 299,010 new cases estimated for 2024, representing 14.9% of all new cancer diagnoses. Those initially diagnosed with distant metastatic disease have a reported 5-year survival rate of 36.6%, making immediate and effective systemic intervention essential [1]. Testosterone suppression is the backbone of treatment for metastatic prostate cancer, and the addition of second-generation androgen receptor blockades as well as taxane-based chemotherapy to this backbone have been associated with improved survival.

PSMA-Lu177, a novel radionucleotide therapy, has recent FDA approval for use in mCRPC with PSMA-avid metastases on PET scan after disease progression on second generation androgen receptor blockade, as well as taxane-based chemotherapy [2]. Primary resistance to ADT, second generation anti-androgens, and chemotherapy, however, is rare. Just 4% of all prostate cancers are diagnosed as de novo metastatic disease, and such cases have a shorter time course to castrate-resistance [3].

Here we present a case of an elderly patient with mCRPC and primary resistance to ADT, second generation androgen receptor blockade, and docetaxel chemotherapy, who experienced a rapid and significant response to PSMA-Lu177.

### Case

**Fig. 1:**
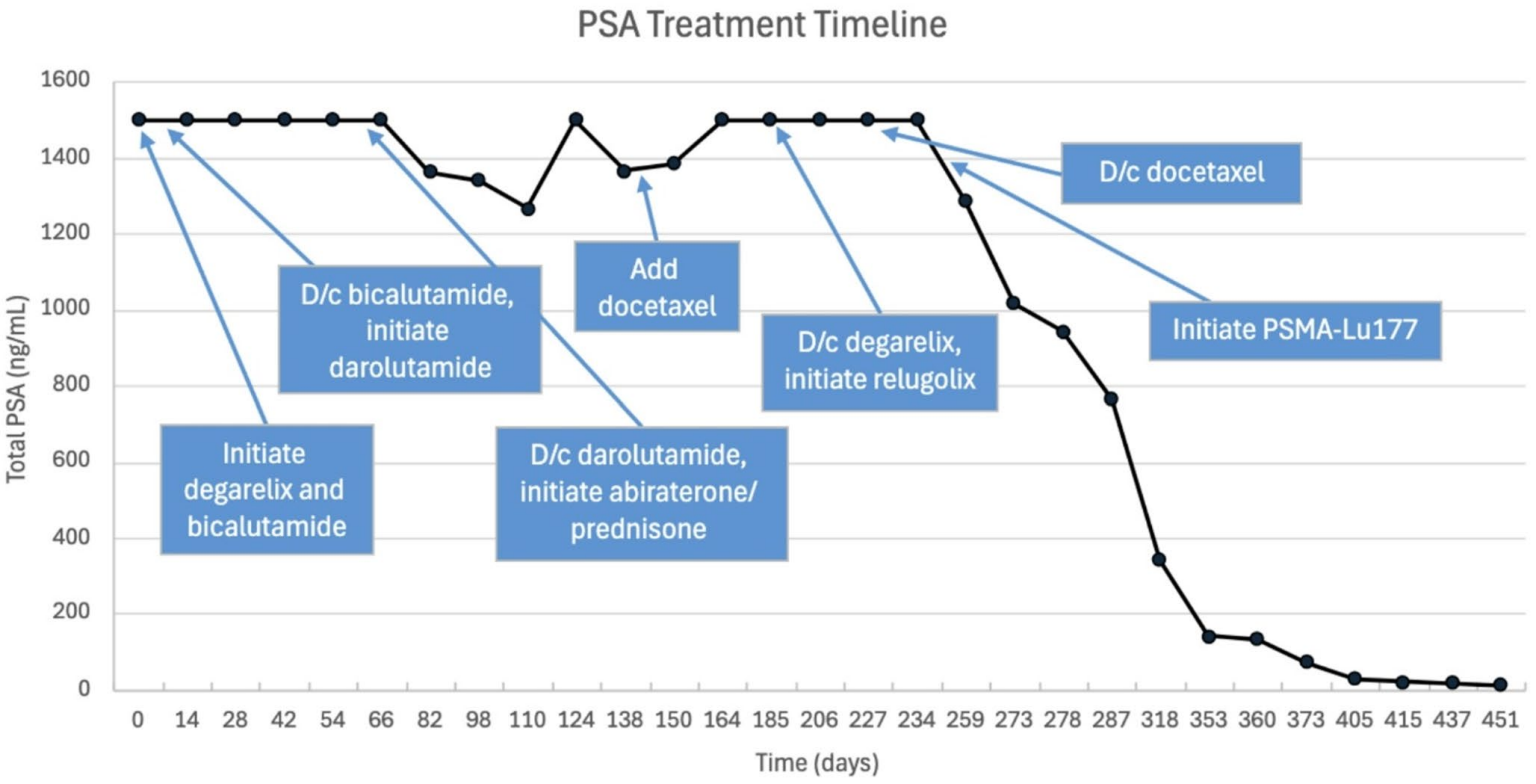
PSA trend along the patient’s treatment timeline with day 0 as the initiation of systemic therapy. Lack of response to ADT, second generation anti-androgen, and 4 cycles of docetaxel shown. Significant PSA decline demonstrated after 5 cycles of PSMA-Lu177. (D/c: discontinued)

**Fig. 2:**
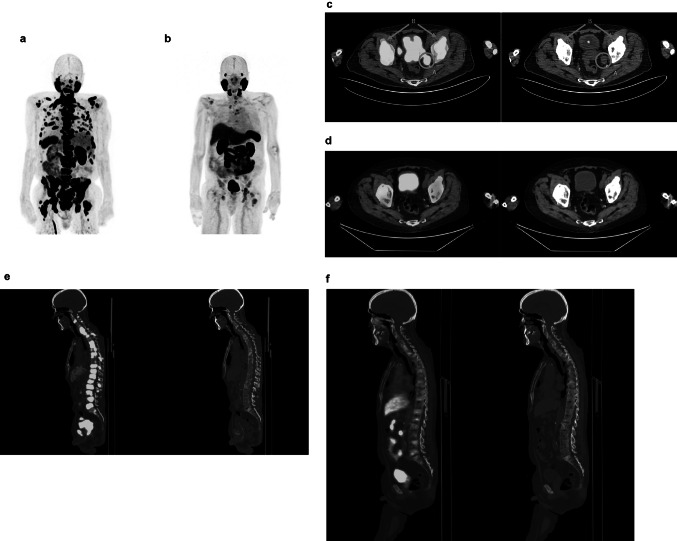
a) 3D MIP of a Gallium-68 PSMA PET scan at time of diagnosis showing numerous foci of PSMA avid uptake within the prostate mass, supraclavicular, retroperitoneal, and pelvic lymph nodes, and the axial and proximal appendicular skeleton. b) 3D MIP of a Gallium-68 PSMA PET scan 16 months following initial scan, after treatment with PSMA-Lu177, showing marked improvement of previously visualized areas of PSMA avid uptake within the prostate mass, supraclavicular, retroperitoneal, and pelvic lymph nodes, and the axial and proximal appendicular skeleton.c) Axial PSMA PET/CT (left) and CT soft tissue window (right) at time of diagnosis showing avid PSMA uptake within a 2 cm short axis left pelvic sidewall lymph node (A, circled) and the bilateral pelvic bones (B, arrows).d) Axial PSMA PET/CT (left) and CT soft tissue window (right) 16 months after initial scan showing resolution of the previously visualized 2 cm short axis left pelvic sidewall lymph node and improvement of PSMA uptake within the bilateral pelvic bones.e) Sagittal PSMA PET/CT (left) and CT bone window (right) at time of diagnosis showing diffuse avid PSMA uptake within the spinal vertebral bodies and posterior elements with associated mixed lytic and sclerotic lesions, reflecting diffuse osseous metastatic disease.f) Sagittal PSMA PET/CT (left) and CT bone window (right) 16 months following initial scan showing interval decrease in intensity of PSMA uptake within the spinal vertebral bodies and posterior elements with associated persistent mixed lytic and sclerotic lesions.

Figure.1We present a case of a 90-year-old Caucasian male with de novo high-volume metastatic prostate adenocarcinoma diagnosed after he presented to the ED for evaluation of bilateral hip and flank pain. Diagnostic imaging revealed an irregularly enlarged prostate and seminal vesicles, with multiple sites of lymphadenopathy and diffuse mixed sclerotic and lytic osseous metastases. PSA at this time was 1420 ng/mL. A PSMA/PET scan revealed widespread metastatic disease involving the appendicular and axial skeleton (Fig. 2a, c and e), and prostate biopsy confirmed diagnosis of Gleason 5 + 4 prostate adenocarcinoma involving 80% of 3/3 cores. Next-generation sequencing (NGS) on prostate biopsy showed PTEN LOF, ZFHX LOF, TMB 2.6, with MSI stable, and HRD not detected.

Table 1 summarizes the patient’s treatment course. He was initially started on degarelix and bicalutamide 11 days following initial diagnosis, and was then quickly switched to darolutamide. Darolutamide was given at a lower dose, 300 mg twice daily, secondary to renal impairment. Due to his chronic kidney disease and poor functional status, he was not offered chemotherapy at this time. PSA at the time of initiating treatment was > 1500 ng/mL, and total testosterone levels were 221 ng/dL.

**Table 1:** Timeline of treatment course and response to therapy

Timepoint/Event	Current therapy	PSA (ng/mL)	Testosterone (ng/dL)	Creatinine (mg/dL)	Notes	ECOG status	Change in therapy
**Initial diagnosis**	-	1420	-	-	Diffuse osseous metastases	2	-
**11 days following diagnosis**	Degarelix + bicalutamide initiated → quickly switched to darolutamide 300 mg twice daily (due to renal impairment)	> 1500	221	2.1	Chemotherapy not offered as poor candidate (CKD, comorbidities)	2	-
**2 months post treatment start**	Degarelix + 300 mg twice daily darolutamide (reduced dose)	> 1500	< 10	1.7	Unchanged PSA representing primary resistance to ADT + second-generation anti-androgens	2	Darolutamide discontinued, began **abiraterone/prednisone**, received palliative radiation to pelvis
**5 months post treatment start**	Degarelix + abiraterone/prednisone 1000 mg daily/5 mg twice daily	1364	57	1.8	PSA declined after 3 weeks of starting new regimen, but plateaued 2 months later at 1364; clinical/performance status improvement	1	Began reduced dose **docetaxel** (60 mg/m²), switched from degarelix to **relugolix**
**8 months post treatment start**	Relugolix + abiraterone/prednisone 1000 mg daily/5 mg twice daily + docetaxel 60 mg/m² (reduced dose)	1364	25	1.7	Poor PSA response/disease progression after 4 cycles of docetaxel, PSMA/PET showing widespread disease with PSMA expression	1	Discontinued docetaxel; began full-dose **PSMA-Lu177**
**13 months post treatment start**	Relugolix + abiraterone/prednisone 1000 mg daily/5 mg twice daily + PSMA-Lu177	2.6	38	2	5 cycles of PSMA-Lu177 well-tolerated despite CKD	1	-

Unfortunately, PSA remained unchanged 2 months later, indicating a primary resistance to ADT and second-generation anti-androgens. As a result of the lack of PSA response, significant fatigue, and loss of appetite on this regimen, as well as the inability to recommend full dose darolutamide due to a creatinine of 1.7 mg/dL, he discontinued darolutamide and began abiraterone/prednisone. After 3 weeks, the PSA began to slowly decline, however plateaued 2 months afterward at 1364 ng/mL. Given the insignificant change in PSA along with improved clinical/performance status (now ECOG 1, from baseline of ECOG 2), he was offered chemotherapy, beginning reduced-dose docetaxel at 60mg/m^2^. He was also switched from degarelix injections to the oral form, relugolix.

After 3 months of reduced dose docetaxel, he again showed no significant PSA response amidst continued clinical improvement. Repeat PSMA/PET revealed significant metastatic disease with expression of the PSMA receptor. The patient then discontinued docetaxel after 4 cycles and was recommended full dose PSMA-Lu177 therapy. Although PSMA-Lu177 may worsen renal function due to its renal excretion, his lack of response to multiple lines of treatment and overall poor prognosis led to the decision to initiate PSMA-Lu177, despite suboptimal renal function (creatinine 1.7 mg/dL, CrCl 29.5). Surprisingly, following the first cycle of PSMA-Lu177, his PSA sharply declined, most recently at 2.6 ng/mL after 5 cycles. Figure 2b and d, and 2f also showcase repeat scans done 7 months following PSMA-Lu177 initiation with marked improvement. 

He also tolerated PSMA-Lu177 very well despite his advanced age. His renal function on PSMA-Lu177 remained suboptimal but stable, with his most recent creatinine at 2.0 mg/dL. Of note, the patient reported urinary incontinence after his Foley catheter removal during PSMA-Lu177 treatment. He was counseled on the importance of preventing incontinence while on PSMA-Lu177 to avoid unwanted radiation exposure, and required a new Foley catheter for the first week of each PSMA-Lu177 treatment.

## Discussion

As reported in this case, our patient’s prostate cancer showed primary resistance to multiple lines of therapy including ADT, second generation anti-androgens, and chemotherapy, evidenced by the lack of significant PSA response. We defined primary resistance to ADT as the absence of PSA response 2 months following ADT initiation. Primary resistance to second-generation anti-androgens is also defined as an absence of PSA reduction/treatment failure at 3 months [4]. Only with the initiation of PSMA-Lu177 was there a dramatic and sharp decline in our patient’s PSA levels, indicating the utility of PSMA-Lu177 in the setting of PSMA-positive mCRPC patients post-docetaxel therapy, and the possible use of PSA as a marker of treatment response despite castrate-resistance. Moreover, primary resistance to both ADT and anti-androgen therapy is unusual and not fully understood. Pre-clinical studies have shown pre-existing castrate-resistant like prostate cancer cells, which suggests a separate mechanism than adapted resistance following ADT/anti-androgen therapy [5].

Table 2 summarizes three related reports of significant PSA response following PSMA-Lu177 in mCRPC patients. All three patients (cases 1, 2, and 3) demonstrated a > 90% reduction in PSA values post-treatment. Two patients (case 2 and 3) had significant renal impairment, however tolerated PSMA-Lu177 without evidence of nephrotoxicity.

**Table 2:** Reports of exceptional PSA response following PSMA-Lu177 in mCRPC: literature review [6–8]

Case	Age	Primary cancer	PSA at time of PSMA-Lu177 initiation (ng/mL)	PSA post PSMA-Lu177 (ng/mL)	Cycles of PSMA-Lu177	Baseline renal characteristics
1	65	mCRPC	57.7	1.3	2	-
2	68	mCRPC	4667	11.35	4	Post-transplant
3	78	mCRPC	1000	0.84	4	MAG3 renogram with 13% R kidney contribution to total function
4 (our case)	90	mCRPC	>1500	2.6	5	Creatinine 1.7 mg/dL CrCl 29.5

There have been multiple studies aimed at determining the next line of therapy for patients with mCRPC post-docetaxel therapy. Notably, the TROPIC trial demonstrated cabazitaxel’s effectiveness with a median overall survival (OS) of 15.1 months, and a progression free survival (PFS) of 2.8 months [9]. In the AFFIRM trial, enzalutamide therapy showed a median OS of 18.4 months and a PFS of 8.3 months [10]. In addition, the COU-AA-301 trial revealed abiraterone treatment associated with an OS of 14.8 months and a PFS of 5.6 [11]. As these treatment options have not been compared head-to-head, the sequence of therapy is often left to individual physician judgment and expertise [12]. It is important to note, however, that participants in both AFFIRM and COU-AA-301 trials did not receive prior androgen receptor pathway inhibitors (ARPI), due to the unsuccessful nature of sequencing ARPI in mCRPC.

As recently as 2022, PSMA-Lu177 became FDA approved for the treatment of PSMA-positive mCRPC in patients who have been treated with androgen receptor blockers and taxane-based chemotherapy [13]. In the VISION trial which placed PSMA-Lu177 on the mainstage, the investigators demonstrated a median OS of 15.3 and a PFS of 8.7 months [14]. Although PSMA-Lu177 is generally associated with a low incidence of adverse events, it has been reported that patients with mild or moderate renal impairment may be at greater risk of toxicity due to its renal excretion [14, 15]. In our case, the patient displayed borderline moderate renal impairment (creatinine 1.7, CrCl 29.5 prior to initiating PSMA-Lu177), however seemed to tolerate the treatment well, even when considering his advanced age.

Another important consideration in patients with renal impairment and/or urologic pathologies leading to urinary incontinence is the excretion of PSMA-Lu177. The prevalence of urinary incontinence is substantially increased in patients with prostate cancer, and may be due to the effects of radiation, surgery, urinary obstruction, or aging [16]. PSMA-Lu177 is primarily cleared from the body via rapid urinary excretion, and it is cleared in the first 48 h following injection [17]. For this reason, urinary incontinence is a cause for concern in patients undergoing PSMA-Lu177 treatment as it could subject nursing staff, other patients, family members, and other individuals to radiation exposure. As a result, urinary incontinence must be addressed, and careful attention must be paid to these patients during administration, treatment course, and disposal to prevent unwanted radiation exposure.

In conclusion, the results of our case are promising with respect to the future use of PSMA-Lu177 in mCRPC. With its limited side-effect profile and success in this case, there suggests a potential opportunity to consider PSMA-Lu177 earlier in the PSMA-positive mCRPC treatment algorithm, particularly for patients with renal dysfunction and primary resistance to hormone therapy.

## Data Availability

All data supporting the findings of this study are available within the paper and its Supplementary Information.
